# Outcome after extended follow-up in a prospective study of operable breast cancer: key factors and a prognostic index

**DOI:** 10.1038/sj.bjc.6600335

**Published:** 2002-07-15

**Authors:** R A Hawkins, A L Tesdale, R J Prescott, T Forster, M A McIntyre, P Baker, W J L Jack, U Chetty, J M Dixon, M E Killen, M J Hulme, W R Miller

**Affiliations:** University Department of Surgery, Royal Infirmary NHS Trust, Edinburgh EH3 9YW, UK; Medical Statistics Unit, Public Health Sciences, The University of Edinburgh Medical School, Teviot Place, Edinburgh EH8 9AG, UK; Department of Pathology, Western General Hospital NHS Trust, Edinburgh EH4 2XU, UK; University of Birmingham, Birmingham B15 2TH, UK; The Edinburgh Breast Unit, Western General Hospital, Edinburgh EH4 2XU, UK; Edinburgh Breast Unit Research Group, Paderewski Building, Western General Hospital, Edinburgh EH4 2XU, UK

**Keywords:** prognosis, prognostic factors, time-dependent, steroid receptors, prognostic index

## Abstract

In 1990, 215 patients with operable breast cancer were entered into a prospective study of the prognostic significance of five biochemical markers and 15 other factors (pathological/chronological/patient). After a median follow-up of 6.6 years, there were 77 recurrences and 77 deaths (59 breast cancer-related). By univariate analysis, patient outcome related significantly to 13 factors. By multivariate analysis, the most important of nine independent factors were: number of nodes involved, steroid receptors (for oestrogen or progestogen), age, clinical or pathological tumour size and grade. Receptors and grade exerted their influence only in the first 3 years. Progestogen receptors (immunohistochemical) and oestrogen receptors (biochemical) were of similar prognostic significance. The two receptors were correlated (*r*=+0.50, *P*=0.001) and displaced each other from the analytical model but some evidence for the additivity of their prognostic values was seen when their levels were discordant.

*British Journal of Cancer* (2002) **87**, 8–14. doi:10.1038/sj.bjc.6600335 www.bjcancer.com

© 2002 Cancer Research UK

## 

A wide variety of clinical, pathological and biochemical factors are of value in assessing prognosis and therefore in influencing the management of patients with operable breast cancer (Stoll, 1992). In 1990, a prospective trial examined four biochemical factors of reported prognostic value, in a series of consecutive patients in whom sufficient tumour was available analysis. After a median follow-up of 3 years, data were analysed and the findings reported (Hawkins *et al*, 1996).

The data have now been re-analysed at a median follow-up of 6.6 years with inclusion of a fifth biochemical marker and a Prognostic Index has been derived from the key, important influential factors.

## METHODS

Details of the patients entered into the study and the methods employed have been reported previously (Hawkins *et al*, 1996).

### Patients

All women presenting between March 1990 and September 1991 at the Edinburgh Breast Clinic with a unilateral, operable tumour, containing sufficient tissue with >10% cancer cells to permit performance of four biochemical analyses, were included in this study.

Originally 215 patients (from approximately 650 newly diagnosed breast cancers during this period) fulfilled these criteria. One patient had previously undergone surgery for an invasive cancer so this patient was excluded, leaving 214 patients for analysis. One hundred and seventy-two patients received endocrine therapy (167 adjuvant, five neoadjuvant), 37 chemotherapy (33 adjuvant, four neoadjuvant) and seven had no adjuvant systemic therapy. The number of nodes involved (no. nodes +) was known in 190 out of 214 patients from either node sampling or clearance, both of which procedures provide adequate information (Steele *et al*, 1985).

The median age at presentation was 55 years (*n*=214). Median follow-up is now 6.6 years (range 5.3–7.7).

### Tumour histopathology

Tumour (T) histopathology was reviewed by one of us (M McIntyre) for pathological size, tumour grade and histopathological type. Histological grade was assessed using the Bloom and Richardson (1957) system as modified by Elston and Ellis (1991). Histopathological type was classified as previously described (Hawkins *et al*, 1996).

### Determination of the biochemical markers

The methods for determination of oestrogen receptors (ER) and epidermal growth factor receptors (EGFR), cyclic adenosine monophosphate-binding proteins (cAMP-b), cathepsin D (Cath D) and protein concentration in cytosol or membranes have been reported in detail previously (Hawkins *et al*, 1996).

### Determination of progestogen receptor (PgR) protein

Paraffin blocks, derived from the portion of the biopsy originally used for the assay of the four biochemical markers, were sent to the University of Birmingham. Sections were cut, incubated overnight with a 1 : 4 dilution of 0.1 mg ml^−1^ anti-PgR rat monoclonal antibody (Abbott Laboratories) and stained for PgR by a modification of the procedure reported by Soomro and Shousha (1990).

Stained sections were scored, over the whole viable invasive tumour area, for both proportion of cells exhibiting nuclear staining (0–100%) and average staining intensity (0, 1, 2, 3+) in Edinburgh (M McIntyre). A ‘histoscore’ ranging from 0–300, was calculated.

Of the 214 tumour blocks, 204 were assessable for PgR.

### Combination of factors in prognostic indices

For 177 patients, data were available for the calculation of two prognostic indices.

One well-established index (Miller *et al*, 2000), the Nottingham Prognostic Index score (NPI), was calculated according to Haybittle *et al* (1982).

To incorporate the influence of steroid receptor activity, into a prognostic index, receptor levels were scored into four categories, as used by Leake *et al* (2000). These were:


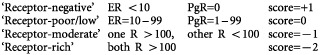


A receptor-modifed prognostic index was then calculated. In calculating a ‘Receptor-modified NPI’, no attempt was made to assess the *optimal* weighting for each factor. Instead, we arbitrarily allowed receptor status (values −2, −1, 0 and +1), which in our data, appears to have approximately the same level of prognostic influence as tumour grade (values 1, 2 and 3), to reduce the NPI by a maximum of two units (high R levels=improving prognosis) or increase it by one unit (R-negative=worsening prognosis).

### Statistical analysis

All continuous variables were entered into the analysis without categorisation or use of cut-offs.

**Figure 2:**
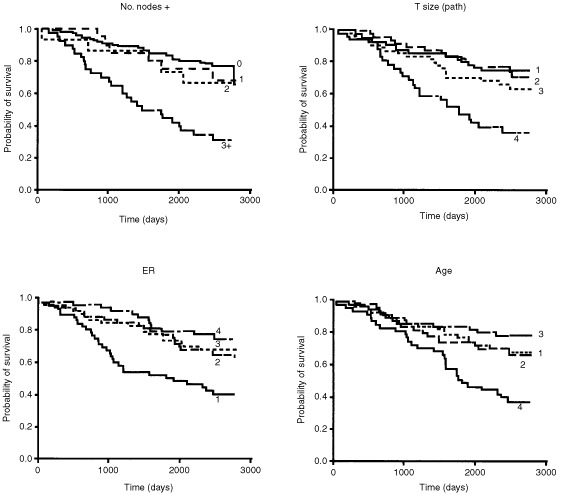
Kaplan–Meier curves for overall survival in 214 patients with operable breast cancer, after division of the patients into quartiles (for Path T size, these were <1.5, ⩾1.5–<2.2, ⩾2.2–3.0 and >3.0 cm; for ER <11, 11–63, 64–164 and >164 fmol mg^−1^ protein and for age, <49, ⩾49–⩽55, 56–63 and >63 years) or four groups (Nodes involved 0, 1, 2 and >3).

**Figure 3:**
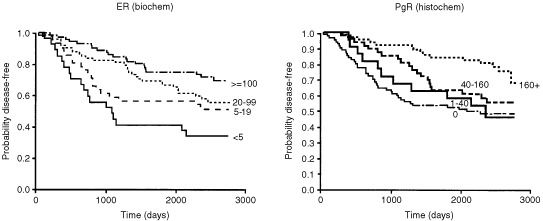
Comparison of the relationships between ER, determined by biochemistry, and PgR, determined by immunohistochemistry, to disease-free survival, using optimised levels.

For graphical presentation, the data were divided into quartiles (tumour size, ER, age – Figure 2

) or categories selected on the basis of prior analyses (number of positive nodes – Figure 2, PgR histoscore – Figure 3

) or experience (ER – Figure 3) and Kaplan–Meier estimates were used to provide survival curves.

Where hazard ratios have been used to indicate the magnitude of a factor's influence, these have been calculated for arbitrarily selected, significant increments (doubling of R concentration, decade increases in age, increase in pathological size by 1 cm, tumour grade by one unit, soluble protein concentration by 1 mg ml^−1^) or categories selected on the basis of preliminary analysis (number of positive nodes).

The inter-relationships between variables were examined by calculation of Spearman's rank correlation coefficients.

The associations of the potential prognostic factors with time to recurrence, disease-free survival and survival were investigated using Cox's proportional hazards models. In these analyses, variables with a skewed distribution (ER, PgR, Cath D and EGFR) were log-transformed. Parameters determined at presentation or surgery but influencing treatment (type of surgery, type of systemic therapy and radiotherapy) were excluded from multivariate analyses.

All prognostic variables were included singly in the models to assess their univariate association with outcome variables (Table 1

**Table 1:**
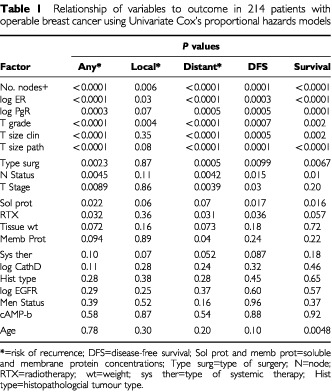
Relationship of variables to outcome in 214 patients with operable breast cancer using Univariate Cox's proportional hazards models

). Variables showing a statistically significant association with at least one outcome variable were then considered in multivariate analyses with allowance for possible time-dependency in the effect of variables on outcome (Table 2

**Table 2:**
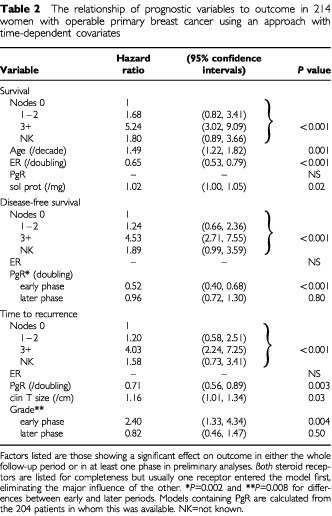
The relationship of prognostic variables to outcome in 214 women with operable primary breast cancer using an approach with time-dependent covariates

). For each significant variable in preliminary analysis, a new, time-dependent covariate was generated to allow the prognostic effect to differ between ‘early’ (first 3 years) and ‘later’ (>3 years) time-periods. A model containing all ‘significant’ variables and their corresponding time-dependent co-variates was then fitted, with a stepwise removal of all non-significant (*P*>0.05) time-dependent variables. The final model contains a mixture of variables, some of which showed a statistically significant difference between their ‘early’ and ‘later’ effects and others which show an overall significant effect, but no time-dependency.

Cox's proportional hazards models were used to test whether modification of the NPI to include the influence of steroid receptors improved the relationship between the index and survival.

## RESULTS

### Patient outcome

Seventy-seven (36%) recurrences and 77 (36%) deaths occurred during follow-up. Forty-seven patients had distant recurrences, 11 loco-regional recurrence only and 19 both. Fifty-nine (77%) deaths were breast cancer-related, the remainder (18) being largely of cardiovascular origin. One hundred and seventy-seven patients were alive after 3 years and available for further analysis of the post 3-year period; 137 were alive at latest follow-up.

### Associations between variables

**Figure 1:**
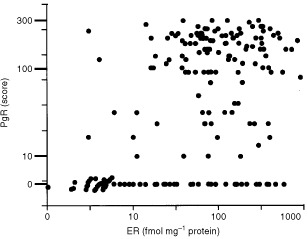
The relationship between ER measured by biochemistry and PgR detected by immunohistochemical staining in 204 patients with operable breast cancer. Spearman's Rank correlation coefficient=+0.50, *P*<0.001.

Significant associations existed between the prognostic variables (see Table 1 in Hawkins *et al*, 1996). Of the newer variables, PgR (any nuclear staining) was detected in 162 out of 204 tumours available for assay. Correlation between PgR and ER levels was highly significant (*P*<0.001) but moderate (Spearman's rank correlation coefficient, *r*=+0.50) and is illustrated in Figure 1

. PgR level was significantly inversely related to EGFR activity (*r*=−0.28, *P*=0.001) and tumour size (clinical, *P*=0.002, *r*=−0.21 and pathological *P*=0.013, *r*=−0.18).

Number of positive nodes correlated with clinical node status (*r*=+0.37, *P*<0.001), T stage (*r*=+0.28, *P*<0.001), clinical T size (*r*=+0.32, *P*<0.001) and pathological T size (*r*=+0.24, *P*=0.001), T grade (*r*=+0.16, *P*=0.025), histopathological T type (*r*=+0.14, *P*=0.04), tissue weight (*r*=+0.26, *P*=0.001) and Cath D activity (*r*=+0.15, *P*=0.026).

### Relationship of variables to outcome by univariate analysis

Thirteen variables were significantly related to at least one outcome measure (Table 1).

For local recurrence, the most important factors were number of positive nodes, T grade and ER activity. Factors related to distant recurrence, disease-free survival and overall survival were very similar except that for overall survival, age was a highly significant factor (*P*=0.005).

Four key factors influenced outcome, number of positive nodes , pathological T size, ER concentration and age and these were divided into quartiles (for nodes involved, four groups were 0, 1, 2 and >3); their effect is illustrated as Kaplan–Meier survival curves in Figure 2. The effect of age related specifically to a poorer survival for the oldest patients dying from all causes (age >63 years) or from breast cancer only (not shown).

### Relationship of variables to outcome by multivariate analysis

The important factors (Table 2) were number of positive nodes, steroid receptors, tumour size and grade, patient age and soluble protein concentration.

The effects of receptors and grade were, however, apparent only during the first 3 years of follow-up.

### Comparative importance of ER and PgR as prognostic factors

Oestregen receptor and PgR activities were the only ‘biochemical’ activities confirmed to be of prognostic significance. The prognostic data obtained from the two measurements are virtually equivalent. This is illustrated in the survival curves in Figure 3: ER was divided according to levels found useful through experience (<5=‘negative’, 5–19=‘poor’, 20–99=‘moderate’, >100=‘rich’, fmol mg^−1^ protein) whilst for PgR, the cut-offs (0, 1–39, 40–160 and >160 histoscore) were selected retrospectively to provide the best separation between survival curves.

There were 33 patients in whom there was some discrepancy between the results of ER and PgR assays (Table 3

**Table 3:**
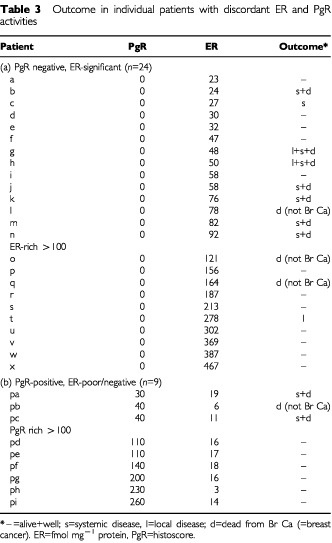
Outcome in individual patients with discordant ER and PgR activities

). Of these, patients with a tumour rich in one receptor (*either* ER *or* PgR) had a better outcome than those with tumours rich in *neither* receptor.

### Combination of prognostic factors as prognostic indices

The Nottingham Prognostic Index (NPI) was calculated for the 177 patients with sufficient data. Increasing levels of the NPI were associated with decreasing percentages of patients surviving (from death of any cause) at 3, 5 or 7 years as demonstrated in Table 4

**Table 4:**
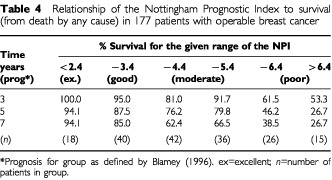
Relationship of the Nottingham Prognostic Index to survival (from death by any cause) in 177 patients with operable breast cancer

.

**Figure 4:**
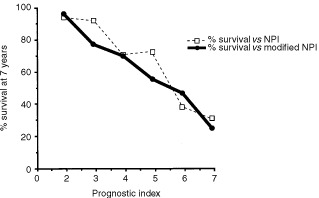
The relationship between the percentage of patients surviving from breast cancer and (a) the Nottingham Prognostic Index (dotted line) and (b) a Steroid receptor-modified NP Index (solid line) for 177 patients with operable breast cancer. Percentage survival values are plotted at the mid-point for each range of indices, e.g. at 2.9 for the group ranging from 2.4–3.4.

When the NPI was modifed to incorporate steroid receptor levels, values ranged from 0.20 to 8.92. The relationship between survival after 7 years and the new, modified index was better, becoming more nearly linear (Figure 4

). This modification produced a statistically significant improvement in the fit (χ^2^=9.9, *P*=0.002).

## DISCUSSION

### Aims of study

The original aim of this study was to identify newer biochemical markers of prognosis which might add to the value of ER (Knight *et al*, 1977; Shek and Godolphin, 1989) to increase the ability to predict outcome in operable breast cancer. However, epidermal growth factor receptors, cathepsin D and cyclic AMP-binding proteins were not related to outcome, by either univariate or multivariate analysis. This may be due to a variety of factors (Hawkins, 1993; Hawkins *et al*, 1996), the population studied, which included mainly larger tumours (sufficient for four biochemical analyses) and consisted of only one-third of all the patients presenting to our Breast Clinic, or to intra-tumoural variations (Barranco *et al*, 1994).

This re-analysis set out to determine the importance of the various factors over a longer period and to determine if the key prognostic factors had changed with time.

### Factors influencing outcome

Three measures of outcome have been considered here: overall survival, disease-free survival and time to recurrence. (The latter relates more specifically to breast cancer than disease-free survival which incorporates mortality unrelated to breast cancer.) Since most of the factors studied are inter-related (e.g. T grade relates to age, T stage, clinical T size, pathological T size, node status, T histopathological type, log ER, and number of positive nodes), then the loss of small numbers of patients from a group of 200 or so (due to missing values) may cause some surprising changes to the factors emerging as ‘significant’ in the final model derived by multivariate analysis. In order to minimise this influence, the analyses were iterated to obtain finally the maximum possible number of patients for whom the key, significant variables were available.

For *overall survival*, the number of nodes involved at either node sampling or node clearance was the most important factor; it displaced clinical node status from the statistical model. Patients with three or more nodes involved had an increased risk of death of over five-fold compared to patients with no nodes involved. ER activity also significantly influenced survival with a decrease in hazard ratio of 0.65 for every doubling of ER level, displacing PgR from the model for this outcome. Age influenced risk of death from all causes (increase in hazard ratio of 1.49 per decade of ageing) and also death due to breast cancer. This is in line with the earlier observations of Adami *et al* (1985). Soluble protein concentration was associated with a slight but significant increase risk of death, as noted by Soreide *et al* (1991); this may be because it accurately reflects tumour cellularity (Hawkins *et al*, 1988).

For *disease-free survival*, number of nodes involved was the key factor with PgR being the only other significant factor. The effect of PgR was time-dependent. This time-dependence of the effect of steroid receptors was apparent, though not statistically significant, in all measures of outcome (overall survival – ER, disease-free survival and time to recurrence – PgR).

For *time to recurrence*, the key factors were number of nodes involved, PgR, tumour grade and clinical size (Table 2).

However, the effect of grade was time-dependent, having its influence only in the early period.

### Role of steroid receptors

Steroid receptors were of prognostic significance only within the first 3 years of follow-up. The time-dependency has been noted previously (Saez *et al*, 1983; Pichon *et al*, 1996). It may explain why, in studies of patients with large, operable tumours treated by neoadjuvant therapy, short-term response but *not* long-term survival related strongly to ER level (Anderson *et al*, 1989; cf. Cameron *et al*, 1997).

Although 172 of our patients received adjuvant endocrine therapy, a similar trend for improving outcome with increasing ER level was apparent in the remaining 43 patients who did not receive endocrine treatment (data not shown); the influence of receptors may thus be independent of the use of endocrine therapy.

Both ER and PgR were related to outcome with one often displacing the other in different multivariate analyses. The value of immunoreactive PgR protein, confirms earlier findings (Baker *et al*, 1995) but is in contrast to our earlier studies (Hawkins *et al*, 1987; Watson *et al*, 1987) using radioligand-binding assay, which may be less reliable for detecting PgR protein. There was some evidence in the current study for an additivity of the prognostic values of the two receptors: patients with a ‘receptor-rich’ tumour (>100 ‘units’ by *either* assay) had a good prognosis.

This ‘additivity’ may relate to (1) the benefits of conducting the ‘receptor’ assay twice (i.e. ‘in duplicate’), (2) the possibility that two different *modes* of assay complement each other or (3) that each protein has a unique prognostic value.

The findings presented here are broadly in line with those of other studies, though slight differences from some studies may relate to the use of receptors as a discontinuous variable with selection of arbitrary cut-offs, or to pooling of data from several centres with different levels of quality control.

### Combination of factors in a prognostic index

For clinical purposes, it is useful to be able to combine key prognostic factors into a single ‘prognostic index’. The most commonly used, the Nottingham prognostic Index (NPI), incorporates tumour grade, pathological size and number of nodes involved. This index was of prognostic value in the present study (Table 4). However, the discriminatory power of the NPI was only slightly better than that found using numbers of nodes involved alone (data not shown). This could be due to our patients with >3 nodes positive having a much poorer survival than those with 1–2 nodes involved (cf. patients with 1–3 nodes positive being pooled in the NPI). The NPI omits any prognostic contribution from steroid receptor activity. Modification of the NPI by incorporating a ‘correction’ for steroid receptor levels significantly improved the relationship to survival at 7 years (Figure 4).

All prognostic indices reflect estimates of a probability of outcome for which there can be no certainty. In this series, one patient, with a grade-III, receptor-negative tumour of 9.6 cm and 12 nodes involved at presentation, was alive and well when last seen.

### Conclusion

Factors significantly influencing prognosis include number of nodes involved on sampling or clearance, age, tumour size, steroid receptor activity and tumour grade. The effects of steroid receptors and grade are time-dependent. ER and PgR are of equivalent value. Combination of number of involved nodes, tumour grade, tumour size and steroid receptors in a prognostic index provides a more accurate assessment of prognosis for most patients than the NPI alone.
